# Does the Quality of Postpartum Hemorrhage Local Protocols Improve the Identification and Management of Blood Loss after Vaginal Deliveries? A Multicenter Cohort Study

**DOI:** 10.3390/healthcare10060992

**Published:** 2022-05-27

**Authors:** Françoise Vendittelli, Chloé Barasinski, Olivier Rivière, Caroline Da Costa Correia, Catherine Crenn-Hébert, Michel Dreyfus, Anne Legrand, Laurent Gerbaud

**Affiliations:** 1 CNRS, SIGMA Clermont, Institut Pascal, CHU Clermont-Ferrand, Université Clermont Auvergne, F-63000 Clermont-Ferrand, France; cbarasinski@chu-clermontferrand.fr (C.B.); cdacostacorreia@chu-clermontferrand.fr (C.D.C.C.); alegrand@chu-clermontferrand.fr (A.L.); lgerbaud@chu-clermontferrand.fr (L.G.); 2 Réseau de santé en Périnatalité d’Auvergne, CHU de Clermont-Ferrand, Site Estaing, Pôle Femme et Enfant, 1 Place Lucie et Raymond Aubrac, CEDEX 1, 63003 Clermont-Ferrand, France; 3 Audipog, Université Claude Bernard Lyon 1-Laennec, 7, rue Guillaume Paradin, CEDEX 08, 69372 Lyon, France; olivier.riviere@audipog.net (O.R.); catherine.crenn-hebert@aphp.fr (C.C.-H.); 4 APHP, Hôpital Louis Mourier, Colombes, 178 rue de Renouillers, 92700 Caen, France; 5 Departement of Gynécologie-Obstétrique et Médecine de la Reproduction, Normandie Université, UNICAEN, CHU de Caen Normandie, 14000 Caen, France; dreyfus-m@chu-caen.fr; 6 Fédération Française des Réseaux de Santé en Périnatalité (FFRSP), 2 Rude De La Loire, 44200 Nantes, France

**Keywords:** blood transfusion, clinical practice guidelines, postpartum hemorrhage, protocol, severe maternal morbidity, vaginal delivery

## Abstract

Substandard care, which can result from a delayed recognition of the severity of blood loss, can increase maternal morbidity. Our objectives were to assess the incidence of postpartum hemorrhage (PPH) and of second-line procedures in maternity units according to the quality of their PPH protocol. We used a mixed design, a prospective cohort (3442 women with PPH after vaginal delivery; February–July 2011), and an audit of the written protocols (177 French maternity units; September 2010–June 2011). A quality score was calculated for the protocol of each unit. Maternity units were classified into three categories according to this score: category 1 (total score: 0–8), category 2 (9–12.5), and category 3 (>12.5). The PPH incidence (>500 mL) was 3.2%, 3.3% and 4.6% among maternity units in categories 1, 2 and 3, respectively (*p* < 0.0001). The incidence of severe maternal morbidity (surgery and/or artery embolization and/or blood transfusion) was higher among maternity units in category 1 (54.8%; 95% CI: 51.9, 57.7) than in either category 2 (50.1%; 95% CI: 47.8, 52.5) or 3 (38.0%; 95% CI: 33.8, 42.4]) (*p* < 0.0001). The risks of severe maternal morbidity were lower for category 3 than category 1 and 2 (respectively, adjusted RR 0.68, 95% CI 0.60–0.86 and 0.77, 95% CI 0.68–0.87). Finally, maternity units with higher scores identified PPH better and used fewer curative second-line procedures.

## 1. Introduction

Immediate postpartum hemorrhage (PPH) remains a major cause of maternal deaths worldwide [1,2]. Obstetric hemorrhage is also the principal cause of severe maternal morbidity [3], which includes psychological and emotional distress. The incidence rates of mild (≥500 mL and <1000 mL) and severe (≥1000 mL) PPH are reported to be around 6.0% and 1.86% of all deliveries [4], respectively, although they vary widely throughout the world [5]. In a French multicenter cohort study, PPH (>500 mL) incidence was 3.36% (95% CI: 3.25%, 3.47%) and severe PPH (>1000 mL) 1.11% (95% CI: 1.05, 1.18%) after vaginal delivery [6].

Despite a significant reduction in the number of maternal deaths in the world—from an estimated 523,000 in 1990 to 289,000 in 2013—the rate of decline was less than half of what was needed to achieve the Millennium Development Goals target of a three-quarter reduction in the mortality ratio between 1990 and 2015 (https://www.who.int/news-room/fact-sheets/detail/millennium-development-goals-(mdgs) (accessed on 19 May 2022)). For the period 2013–2015, the French maternal mortality ratio was 10.8 per 100,000 live births (95% CI 9.5–12.1), stable compared to 2010–2012 [7]. Hemorrhage-related events are among the most preventable causes of maternal death, to the extent that the most recent French confidential enquiry report on maternal deaths classified most deaths from PPH as preventable or perhaps preventable (84.2%; 16/19 deaths) [7]. Optimizing professional practices to prevent PPH and to manage that which cannot be prevented is an important health research priority worldwide, because early identification and rapid response may reduce maternal morbidity and mortality. In a cross-sectional observational study, we noted differences between the contents of the French 2004 guidelines for the prevention and management of PPH [8] and the self-reported policies of French maternity units [9]. These results, like those of other authors, underline that simply disseminating a policy directive does not suffice to cause the policy to be implemented [10,11,12]. The evidence base supporting decisions about the strategies for guideline dissemination and implementation that are most likely to modify clinical practices remains imperfect [13]. Most authors have studied the impact of multifaceted intervention on the PPH rates or on practices for the prevention, diagnosis and management of PPH [14,15,16,17,18,19,20,21,22], but no compelling evidence has shown that multifaceted interventions are more effective than single-component interventions [23]. One important way to optimize PPH prevention and care may be the translation of evidence-based guidelines into comprehensive maternity unit protocols. Only a few studies have performed audits of the quality of protocols, and they have underlined their suboptimal quality [24,25].

We hypothesized that maternity units with protocols adhering most closely to the national guidelines estimate blood loss better and perform second-line treatments for PPH (artery embolization, surgical procedures and transfusions) less often. Thus, the principal objective of our study was to assess the incidence of PPH (>500 mL) in maternity units according to the quality of their PPH protocols. The secondary objectives were to assess the incidence of mild PPH (>500 mL and ≤1000 mL) and severe PPH (>1000 mL), separately, and the incidence and risk of nonpharmaceutical curative second-line procedures performed for PPH in maternity units, also according to the quality of their protocols. 

## 2. Methods and Materials

### 2.1. Study Design and Setting

In a first step, an observational multicenter audit was conducted to assess the quality of the written protocols of French maternity units for the prevention and management of immediate PPH [25]. In September 2010, we asked 300 maternity units to send the study team a copy of the protocol used in their maternity unit (i.e., specific to their maternity unit or, at minimum, their perinatal network) for PPH prevention and management (by email, no later than June 2011). Two email reminders were sent during this period. We built an audit screening form to assess each unit’s protocol based on 22 criteria highly recommended by the French College of Gynecologists and Obstetricians (CNGOF): 15 for vaginal deliveries (Table S1) and 7 applicable only to caesarean deliveries [25]. These ranged from a clear definition of PPH to the steps for its prevention and management [8]. A glossary accompanied the screening form to prevent information bias during the audit. Two research midwives independently reviewed all protocols. Disagreements were resolved by meetings between the midwives and the first author until consensus was reached. Of the 244 maternity units responding (participation rate of 81.3%), 97.1% had a written protocol. 

In a second step, we conducted a prospective observational PPH incidence study among 182 French maternity units (over 231 eligible maternity units; participation rate of 78.79%). Its methods and results have been detailed elsewhere [6]. The study presented here concerns only vaginal deliveries; caesarean deliveries are excluded because the definition of PPH differed in the HERA study for vaginal (<500 mL in the 24 h after delivery) and caesarean deliveries (>1000 mL). Eligible women had singleton or multiple pregnancies, regardless of parity, gave birth to stillborn or live born babies by vaginal delivery, after a gestation of at least 22 weeks. In each case of PPH, the medical and/or surgical treatment and maternal outcomes were recorded. Blood loss was to be estimated at least visually. Professionals in each unit collected data prospectively for 6 months (1 February–31 July 2011). That study identified 3442 women with vaginal deliveries and PPH.

In the third step, we analyzed only the 177 maternity units that had participated in both components of the HERA study (PPH incidence and protocol audit), described in steps one and two [6,25]. 

### 2.2. Outcome Measures

The principal endpoint was the incidence of PPH (>500 mL). The secondary outcome measures were the incidence of mild PPH (>500 mL and ≤1000 mL) and severe PPH (>1000 mL), separately, as well as the overall incidence of nonpharmaceutical curative second-line procedures performed for PPH (one or more of surgical procedures, arterial embolization, and blood transfusion), and the incidence of each one of these procedures separately. The surgical procedures included B-Lynch or Ho-Cho suture, hypogastric arterial ligation, other vessel ligation, hysterectomy, cervical suture, and suture of vaginal laceration.

### 2.3. Statistical Analysis 

A quality score was calculated based on the 15 criteria for vaginal deliveries. According to the authors’ assessment of each criterion’s clinical importance, a score of 0.5, 1 or 1.5 was attributed to it, or 0 in the absence of any concordance or any protocol (six maternity units had no protocol) (Table S1). The maximum score possible was 15. 

Maternity units were then distributed into three protocol-quality categories based on this score: category 1 (total score of 0–8), category 2 (total score of 9–12.5) and category 3 (score > 12.5). Category 1 comprised the maternity units with a score ≤ 40th percentile, and category 3 those with a score ≥ 90th percentile of all the observed scores. We then assessed the incidence of PPH and of the second-line procedures according to these categories. Additionally, women with a PPH > 500 mL (global PPH) were split into two subgroups: mild PPH (blood loss > 500 mL to 1000 mL or less) and severe PPH (blood loss > 1000 mL).

The percentages are presented with their 95% confidence intervals (95%CI) and were compared with a Chi2 statistical test; means were compared with an Anova test. Crude relative risks (RRs) were calculated with their 95% confidence intervals (95% CI). A generalized linear model using Poisson regression was applied to adjust for confounding factors for each of our outcome measures, and adjusted relative risks (aRR) were calculated with their 95% CI. Significance was set at 0.05. Data collection and analysis were performed with SAS software (version 9.4, SAS Institute, Cary, NC, USA, 2002–2012).

## 3. Results

### 3.1. Maternity Unit Characteristics

During the study period, the participating units managed 3442 women with a PPH after a vaginal delivery (*n* = 2728 women with a mild PPH > 500 mL and ≤1000 mL and 714 women with a severe PPH > 1000 mL). Among the 177 maternity units, 16.4% were level III and 8.5% university hospitals (Table 1). Level II units, although only 38% of all participating maternity wards, accounted for nearly two thirds of those ranked in category 3 (Table 1). 

The caesarean rate was 19.6% (95% CI: 19.3, 19.8) in all participating maternity units and 20.3% (95% CI: 19.9, 20.7) in category 1, 19.3% (95% CI: 19.0, 19.6) in category 2, and 18.3% (95% CI: 17.6, 18.9) in category 3.

### 3.2. Patient Characteristics 

During the study period, from 1 February to 31 July 2011, there were 101,339 spontaneous or instrumental vaginal deliveries in the participating maternity units. Among these women, the incidence of PPH was 3.4% (3442/101 339; 95% CI: 3.3, 3.5) and of severe PPH, 0.7% (714/101 339; 95% CI: 0.6, 0.8). In this cohort, 96.8% of infants were singletons, born at a mean gestational age of 39.6 (SD 2.3) weeks; 15.6% of the women were aged 35 years or older, and 79.6% had an actively managed third stage of labor (Table 2). Blood loss was estimated only by visual estimation in 21.4% of PPH (Table 2). Patient characteristics did not differ between the three categories, except for estimated blood loss, where objective estimates were most frequent in category 3 (Table 2). The single maternal death reported was due to an amniotic fluid embolism. 

### 3.3. Incidence of PPH and of Second-Line Treatment According to Audit Criteria 

The incidence rates of PPH and second-line procedures by the presence of a protocol and by each criterion of the audit rubric are reported in Table 3, and Supporting Information 2 and 3 (Tables S2 and S3). The incidence of PPH was lower when the maternity unit used a local protocol (*p* < 0.0001) (Table 3). These incidence rates varied according to the audit rubric criterion (Tables S2 and S3). For example, the incidence of surgical procedures was lower (*p* = 0.0007) and transfusions more frequent (*p* = 0.03) when the protocol called for recording blood loss volume in the medical file. 

### 3.4. Incidence of PPH and Incidence and Risks of Second-Line Treatment According to Maternity Unit Quality Score Categories 

The global incidence of PPH (exceeding 500 mL) was higher in maternity units in category 3 (4.6%; 95% CI: 4.2, 5.0) than in those in categories 1 (3.2%; 95% CI: 3.0, 3.4) and 2 (3.3%; 95% CI: 3.1, 3.4) (*p* < 0.0001), as was the incidence of mild PPH (3.6%; 95% CI: 3.3, 4.2) compared with categories 1 (2.5%; 95% CI: 2.4, 2.7) and 2 (2.6%; 95% CI: 2.5, 2.8) (*p* < 0.0001). Similarly, the incidence of severe PPH (>1000 mL) was also higher in category 3 (1.0%; 95% CI: 0.9, 1.2]) than in categories 1 (0.7%; 95% CI: 0.6, 0.8) and 2 (0.6%; 95% CI: 0.5, 0.7) (*p* < 0.0001).

The incidence of severe maternal morbidity, on the other hand, was higher among maternity units in category 1 (54.8%; 95% CI: 51.9, 57.7) than in either category 2 (50.1%; 95% CI: 47.8, 52.5) or 3 (38.0%; 95% CI: 33.8, 42.4]) (Table 4). The crude RR for severe maternal morbidity, defined as “use of second-line techniques (surgery, artery embolization, or transfusion)” was 31% lower in category 3 than in category 1 and 24% lower than in category 2 (Table 4). After taking maternity unit levels and blood loss estimates into account, the adjusted risk of severe maternal morbidity remained significantly lower in maternity units in category 3 than in categories 1 (aRR 0.68; 95% CI: 0.60, 0.86) and 2 (aRR 0.77; 95% CI: 0.68, 0.87) (Table 4). 

The incidence of surgical procedures alone was also lowest in the category 3 units (*p* < 0.0001). The adjusted risk of a surgical procedure was reduced by 39% in category 3 compared with category 1 and by 22% compared with category 2. We stressed that embolization and transfusion were less frequent in category 1 than in the others, but the adjusted risks did not differ statistically (Table 4). 

We also observed a less frequent use of second-line techniques in category 3, regardless of PPH severity (mild or severe PPH) (Table 4). All adjusted risks for mild and severe PPH are described in Table 4. 

## 4. Discussion

### 4.1. Statement of Principal Findings

We observed a higher incidence of PPH and less frequent use of second-line treatments in maternity units in category 3, those with a PPH protocol that corresponded most closely to the national guidelines.

### 4.2. Strengths and Limitations

The first strength of this work is its originality; to our knowledge, no study of national guidelines has assessed the impact of the quality of protocol contents on maternal morbidity among a large number of maternity units. Moreover, to construct the scale used to audit protocol quality, the authors relied on the French guidelines, which all French birth professionals are supposed to know, as they have been widely disseminated both in French journals, during the annual nationwide conferences of these professionals, CNGOF in particular, and on the CNGOF web site. The second strength of this study is its epidemiological rigor. Maternity units belonging to the regional perinatal networks that agreed to support the HERA study volunteered to collect and record PPH cases prospectively [6]. Two research midwives trained in audit performance conducted the clinical audit. Of the 182 maternity units that participated in the HERA incidence study [6], 177 also participated in the second component of HERA, auditing the presence and quality of the contents of the protocols that these units used (for a participation rate of 97.25%). This study covered 101,339 of all 309,532 French vaginal deliveries, and so accounted for around 32.7% of the vaginal deliveries during the 6-month study period in 2011. This large database has contributed to the continuing debate on the best ways of addressing PPH and guidelines in high-income countries where it remains too often a preventable cause of death.

The first limitation of this study is that caesarean deliveries were not included because of the different definitions of immediate PPH for vaginal and caesarean deliveries in the guidelines then in effect and thus in the HERA study: blood loss exceeding 500 mL for vaginal deliveries and exceeding 1000 mL for caesareans. Since then, the updated 2014 French guidelines have clarified the definition of PPH for all deliveries (≥500 mL) [26]. The second study limitation is that we unfortunately did not cover all French maternity units. In the HERA incidence study, private maternity units participated at a lower rate than the other types of hospitals, as did the units with the fewest deliveries [6]; these two groups overlapped, since most of the private maternity units in France are small. The slightly smaller number of maternity units participating in this study than in the HERA incidence study [6] did not modify the overall incidence rate of PPH after vaginal births in the entire cohort (3.40% in this study vs. 3.36% in the complete cohort). The last limitation is that the HERA study database does not contain data for the women without immediate PPH, so that blood loss could not be estimated for them. It is accordingly impossible to take into account the blood loss estimation to calculate an adjusted risk for PPH. 

### 4.3. Interpretation within the Context of the Wider Literature

In this study, as in that by Bailit et al. [14], the existence of a protocol—either local or that of a regional network—was not associated with a reduction in the use of second-line treatments for PPH. Nonetheless, the simple presence of a protocol is not what is relevant, since its presence says nothing about its quality. Some of the 15 parameters used in this audit may play a more important role in blood loss estimation and second-line PPH treatment than others, as we can see from the Supporting information (Tables S2 and S3). 

We found a higher incidence and risk of PPH and a lower incidence and adjusted risk of surgical procedures among the maternity units in category 3, i.e., the units with the highest quality protocols, than among the other units, although neither maternal characteristics nor pre- and postpartum hemoglobin levels differed between the categories. The multilevel analysis took into account the maternity unit level and the type of blood loss estimation, except for the adjusted risk of PPH. We did not include in our multilevel analysis maternity ward size, because it is highly correlated with its level. This result confirmed our initial hypothesis, as logically expected, because those with a high-quality protocol were probably more aware than those in other categories of the importance of identifying and quantifying blood loss and were, therefore, able to manage PPH earlier. Single-center studies and those with small numbers of participating hospitals that have examined the presence of protocols report contradictory results about the incidence of PPH, including severe PPH requiring second-line treatment [15,27,28,29]. It is impossible to know if the results observed in these studies were causally linked to the drafting of local clinical guidelines, as their intervention also included theoretical and training sessions [15]. As in our work, the single-center studies observed lower maternal morbidity [29,30]. In our study, unlike that by Shields et al. [22], blood transfusions were less frequent among the category 1 maternity units. Shields et al. audited records and processes to assess compliance with PPH protocol recommendations (*n* = 29 hospital units; 32,052 deliveries) and found that the application of a standardized method for addressing maternal hemorrhage significantly reduced maternal morbidity, measured by the need for maternal transfusion (−25.9%; *p* < 0.01) but not peripartum hysterectomy (−14.8%; *p* = 0.2) [22].

### 4.4. Implications for Policy, Practice and Research

Substandard care, which can result from a lack of familiarity with national guidelines and delayed recognition of the severity of blood loss, can increase maternal mortality and morbidity. Our study shows that the quality of the contents of the protocols used by maternity units may well be involved in improving blood loss estimates, which could then reduce maternal morbidity by reducing second-line surgical treatments. Invasive treatments for PPH should be considered as quality markers, or more precisely, indicators of missed opportunities to prevent PPH and/or manage it optimally and thus important keys for preventing severe maternal morbidity.

## 5. Conclusions

Maternity units with the highest quality scores had a higher incidence and risk of PPH, probably due to their better identification of blood loss, as well as fewer secondary procedures that involved notable levels of maternal morbidity. 

The delivery of high-quality patient care is a complex process that demands effective and efficient collaboration by health care professionals. A high-quality protocol can improve the collaboration within the perinatal team, while, as we know, poor collaboration between professionals can aggravate the problems of women with PPH. New research should focus on the improvement of compliance with PPH protocols while taking into account the barriers to change among care providers. 

## Figures and Tables

**Table 1:** Comparison of the characteristics of participating French maternity units overall and according to the protocol-quality category.

	All Maternity Units *n* = 177%	Category 1 Score [0–8] *n* = 79%	Category 2 Score [9–12.5] *n* = 84%	Category 3 Score [>12.5] *n* = 14%	*p* Value
Total no. deliveries ^1^					
<250	7.9	8.9	7.1	7.1	0.04
250–749	58.2	69.6	48.8	50.0	
≥750	42.9	21.5	44.1	42.9	
Level of care ^2^					
Level I	45.2	55.7	40.5	14.3	0.02
Level II	38.4	34.2	38.1	64.3	
Level III	16.4	10.1	21.4	21.4	
Type of facility					
University hospital	8.5	6.3	9.5	14.3	0.15
General hospital	65.5	59.5	69.1	78.6	
Private hospital	26.0	34.2	21.4	7.1	

^1^ Delivery during the 6-month study period. ^2^ Level I: no neonatology department. Level II: presence of a department of neonatology and special care in the same building or immediate proximity to the site of delivery. Level III: neonatal intensive care present in the same building (in addition to neonatology units) or immediate proximity to the delivery room.

**Table 2:** Description of medical data of women who had a PPH after a vaginal birth, overall and according to the protocol-quality category.

	Women with PPH in All Maternity Units *n* = 3442 % [Mean (SD)]	Category 1 Score [0–8] *n* = 1162% [Mean (SD)]	Category 2 Score [9–12.5] *n* = 1770% [Mean (SD)]	Category 3 Score [>12.5] *n* = 510% [Mean (SD)]	*p* Value
Term delivery (weeks)	*n* = 3420 [39.6 (2.3)]	*n* = 1154 [39.7 (2.1)]	*n* = 1758 [39.6 (2.3)]	*n* = 508 [39.7 (2.3)]	0.10
Singletons	96.8	97.5	96.5	96.1	0.20
Women’s age	*n* = 3390	*n* = 1136	*n* = 1746	*n* = 508	
≤18 years	0.7	0.6	0.7	0.8	0.30
>18–35 years	83.7	85.6	82.5	83.9	
≥35 years	15.6	13.8	16.8	15.4	
Hb level before delivery (g/dL)	*n* = 3291 [11.9 (1.1)]	*n* = 1076 [11.9 (1.1)]	*n* = 1712 [11.9 (1.1)]	*n* = 503 [12.0 (1.1)]	0.15
Lowest postpartum Hb level (g/dL)	*n* = 3125 [9.0 (1.6)]	*n* = 1050 [9.0 (1.5)]	*n* = 1608 [8.9 (1.6)]	*n* = 467 [9.2 (1.5)]	0.02
Total estimated blood loss (mL)	[895 (461)]	[909.8 (429.2)]	[883.5 (473.4)]	[896.8 (485.7)]	0.33
Estimated blood loss ^2^	*n* = 3420	*n* = 1148	*n* = 1762	*n* = 510	
Bag	90.3	87.4	90.5	96.5	<0.0001
weighed	14.9	11.4	16.2	18.2	0.0001
Only visual measurement	21.4	23.8	20.8	17.8	0.02
Active management of the third stage of labor ^1^	79.6	80.1	80.0	77.4	0.40

^1^ Active management of the third stage of labor was defined as the use of uterotonic agents after childbirth. ^2^ To participate in the study, blood loss had to be estimated at minimum visually but additional modes of estimation used in the maternity units were also considered. The estimate of blood loss could require the combination of various measurement methods, such as visual estimation or bag and weighing compresses, etc.

**Table 3:** Incidence of global PPH (>500 mL), transfusion of packed red blood cells, surgical procedures, artery embolization among women with a PPH, according to the presence of a local or perinatal network protocol.

Type of PPH Protocol	N ^1^	All PPH Incidence ^2^ % (95% CI) *n* = 101,339 ^3^	*p*	Surgical Procedures % (95% CI) *n* = 3442 ^4^	*p*	Blood Transfusion	*p*	Radiologic Artery Embolization	*p*
						% (95% CI) *n* = 3442 ^4^		% (95% CI) *n* = 3442 ^4^	
Local	177	101,339		3442		3442		3442	
Yes	131	3.3 (3.2, 3.4)	<0.0001	42.0 (40.0, 43.9)	0.33	13.0 (11.7, 14.4)	0.24	2.9 (2.3, 3.6)	0.9
No	46	3.7 (3.5, 3.9)		43.8 (40.6, 47.1)		11.5 (9.5, 13.8)		2.8 (1.8, 4.1)	
Network ^5^	177	101,339		3442		3442		3442	
Yes	40	3.9 (3.7, 4.2)	<0.0001	44.0 (40.7, 47.4)	0.28	11.1 (9.1, 13.4)	0.12	2.7 (1.7, 4.0)	0.7
No	137	3.3 (3.1, 3.4)		42.0 (40.1, 43.9)		13.1 (11.8, 14.5)		2.9 (2.3, 3.7)	

^1^ Number of maternity units with concordant criteria. ^2^ PPH defined as >500 mL. ^3^ *n* = women with a vaginal delivery. ^4^ *n* = women with a PPH after vaginal delivery. ^5^ For maternity units with a local and regional (network) protocol, only the local protocol was considered.

**Table 4:** Incidences and risks of second-line treatments according to protocol-quality category and PPH severity (global, mild, and severe).

	Category 1 % (95% CI)	Category 2 % (95% CI)	Category 3 % (95% CI)	*p*	RR1 ^1^ and 2 ^2^ (95% CI)	aRR1 ^3^ and 2 ^4^ (95% CI)
**Global PPH** (>500 mL)	***n* = 1162**	***n* = 1770**	***n* = 510**			
Severe maternal morbidity ^5^	54.8 (51.9, 57.7)	50.1 (47.8, 52.5)	38.0 (33.8, 42.4)	<0.0001	0.69 (0.61, 0.78) ^1^ 0.76 [0.67, 0.86] ^2^	0.68 (0.60, 0.86) ^3^ 0.77 (0.68, 0.87) ^4^
Surgical procedures and/or artery embolization	50.3 (47.3, 53.2)	43.6 (41.2, 45.9)	33.1 (29.1, 37.4)	<0.0001	0.66 (0.56, 0.76) ^1^ 0.76 (0.67, 0.87) ^2^	0.64 (0.55, 0.74) ^3^ 0.78 (0.68, 0.90) ^4^
Surgical procedures	49.4 (46.5, 52.3)	41.2 (38.9, 43.6)	31.0 (27.0, 35.2)	<0.0001	0.63 (0.54, 0.72) ^1^ 0.75 (0.65, 0.86) ^2^	0.61 (0.52, 0.71) ^3^ 0.78 (0.67, 0.90) ^4^
Radiologic artery embolization	1.7 (1.1, 2.7)	3.8 (3.0, 4.8)	2.4 (1.2, 4.1)	0.004	1.37 (0.67, 2.78) ^1^ 0.62 (0.34, 1.14) ^2^	1.51 (0.71, 2.32) ^3^ 0.62 (0.34, 1.13) ^4^
Transfusion of packed red blood cells	10.6 (8.9, 12.5)	14.5 (12.9, 16.2)	10.8 (8.2, 13.8)	0.003	1.02 (0.72, 1.38) ^1^ 0.75 (0.57, 0.98) ^2^	1.11 (0.80, 1.54) ^3^ 0.72 (0.54, 0.95) ^4^
**Mild PPH** (<500 mL and ≤ 1000 mL)	***n* = 913**	***n* = 1420**	***n* = 395**			
Severe maternal morbidity ^5^	53.9 (50.6, 57.2)	46.3 (43.7, 48.9)	36.0 (31.2, 40.9)	<0.0001	0.67 (0.58, 0.77) ^1^ 0.77 (0.67, 0.90) ^2^	0.65 (0.56, 0.76) ^3^ 0.80 (0.69, 0.93) ^4^
Surgical procedures and/or artery embolization	52.1 (48.7, 55.4)	42.4 (39.8, 45.0)	32.9 (28.3, 37.8)	<0.0001	0.66 (0.57, 0.77) ^1^ 0.79 (0.68, 0.92) ^2^	0.65 (0.55, 0.76) ^3^ 0.82 (0.70, 0.96) ^4^
Surgical procedures	51.0 (47.7, 54.3)	42.5 (39.9, 45.1)	33.7 (29.0, 38.6)	<0.0001	0.65 (0.56, 0.76 ^1^ 0.79 (0.68, 0.92) ^2^	0.64 (0.55, 0.76) ^3^ 0.82 (0.70, 0.96) ^4^
Radiologic artery embolization	1.3 (0.8, 2.4)	1.7 (1.1, 2.5)	0.8 (0.2, 2.2)	0.36	0.58 (0.16, 2.04) ^1^ 0.45 (0.14, 1.48) ^2^	0.64 (0.17, 2.37) ^3^ 0.50 (0.15, 1.67) ^4^
Transfusion of packed red blood cells	6.8 (5.5, 9.0)	7.3 (6.0, 8.8)	4.8 (2.9, 7.4)	0.21	0.71 (0.43, 1.17) ^1^ 0.66 (0.41, 1.06) ^2^	0.72 (0.41, 1.24) ^3^ 0.65 (0.40, 1.06) ^4^
**Severe PPH** (>1000 mL)	***n* = 249**	***n* = 350**	***n* = 115**			
Severe maternal morbidity ^5^	58.2 (51.8, 64.4)	65.7 (60.5, 70.7)	45.2 (35.9, 54.8)	0.004	0.77 (0.62, 0.97) ^1^ 0.69 (0.56, 0.85) ^2^	0.77 (0.61, 0.9) ^3^ 0.69 (0.55, 0.86) ^4^
Surgical procedures and/or artery embolization	47.4 (41.1, 53.8)	48.0 (42.7, 53.4)	31.3 (23.0, 40.6)	0.005	0.66 (0.49, 0.89) ^1^ 0.65 (0.49, 0.87) ^2^	0.61 (0.45, 0.84) ^3^ 0.67 (0.50, 0.90) ^4^
Surgical procedures	45.8 (39.5, 52.2)	40.0 (34.8, 45.3)	24.4 (16.8, 33.2)	0.0005	0.53 (0.38, 0.75) ^1^ 0.61 (0.43, 0.86) ^2^	0.49 (0.34, 0.70) ^3^ 0.63 (0.44, 0.90) ^4^
Radiologic artery embolization	3.2 (1.4, 6.2)	12.3 (9.0, 16.2)	7.8 (3.6, 14.3)	0.0004	2.44 (0.96, 6.15) ^1^ 0.64 (0.32, 1.27) ^2^	2.80 (0.92, 8.53) ^3^ 0.62 (0.31, 1.25) ^4^
Transfusion of packed red blood cells	24.5 (19.3, 30.3)	43.4 (38.2, 48.8)	31.3 (23.0, 40.6)	<0.0001	1.28 (0.90, 1.81) ^1^ 0.72 (0.54, 1.00) ^2^	1.67 (1.11, 2.51) ^3^ 0.73 (0.54, 0.98) ^4^

^1^ Crude RR1: category 3 versus 1. ^2^ Crude RR2: category 3 versus 2. ^3^ RR1 adjusted for maternity unit level and for blood loss estimation [objective (bag and/or weighed) vs. subjective assessment (visual estimates)]: category 3 versus 1. ^4^ RR2 adjusted for maternity unit level and for blood loss estimation (objective (bag and/or weighed) vs. subjective assessment (visual estimates)): category 3 versus 2. ^5^ Severe maternal morbidity = Surgical procedures and/or artery embolization and/or blood transfusion of packed red blood cells.

## Data Availability

The data underlying the findings cannot be made freely available because of French legal restrictions. This is because the present study includes variables that, together, could be used to re-identify the participants based on key characteristics and then have access to other personal data. Therefore, the French Data Protection Agency (Commission Nationale de l’Informatique et des Libertés) strictly forbids making such data freely available. However, data of the audit or aggregated data of the cohort can be obtained upon request from the HERA steering research group. Readers may contact the corresponding author to request the data.

## Supplementary Materials

The following supporting information can be downloaded at: https://www.mdpi.com/article/10.3390/healthcare10060992/s1. Table S1. Criteria selected from the French guidelines to assess the quality of local protocols and their concordance score. Table S2. Incidence of PPH (>500 mL), transfusion of packed red blood cells, surgical procedures, and artery embolization according to each audit criterion for PPH prevention. Table S3 Incidence of PPH (>500 mL), transfusion of packed red blood cells, surgical procedures, and artery embolization, according to audit criteria for PPH management.
