# Supplementary material for: Does the Quality of Postpartum Hemorrhage Local Protocols Improve the Identification and Management of Blood Loss after Vaginal Deliveries? A Multicenter Cohort Study
Source: Healthcare (Basel). 2022 May 27;10(6):992. doi: 10.3390/healthcare10060992 (PMC9222306; doi:10.3390/healthcare10060992)
Supplement: Supplementary file 1 [file healthcare-10-00992-s001.zip › healthcare-1724069-supplementary.pdf]

Table S1. Criteria selected from the French guidelines to assess the quality of local protocols and their concordance score

Selected criteria	Maximum score per criterion
**Existence of a local protocol for PPH**	1
**Existence of only a regional protocol**	0.5
**Criteria covered in the protocol:**	
PPH definition[1]	1.5
Useful contacts and telephone numbers[2]	1
Routine active management of the 2rd stage of labor[3]	1
Time of PPH diagnosis recorded	1
Manual removal of the placenta indicated after 30 min[4]	1
Quantitative assessment of blood loss[5]	1
Total volume of blood loss recorded in medical file	1
Initial management for PPH:	
Technical procedures described[6]	1
Pharmacological procedures described[7]	1
Immediate resuscitation described[8]	1
Specific PPH included in medical file[9]	1
Management for persistent PPH:	
Medical management process described[10]	1
Surgical management process described[11]	1

[1]The audit defined agreement for the definition of postpartum hemorrhage by two criteria: the presence of a specific definition, and its adherence to the definition of the French 2004 guidelines (> 500 ml of blood loss, regardless of mode of delivery). The definition of > 500 ml for vaginal delivery and > 1000 ml for caesarean delivery was also considered correct.

[2]The audit defined correspondence for "useful telephone numbers" by the presence of the numbers for the French

Blood Agency and/or the hospital blood bank. If embolization was available onsite, the telephone number of the interventional radiology department also had to be listed.

[3]The audit defined correspondence by the specification of the first-line pharmacologic product to be used, its dose, and the timing of its use.

[4] The audit considered that concordance was absent when the protocol did not specify manual removal of the placenta after 30 minutes in the absence of its spontaneous delivery.

[5]Regardless of how blood loss was estimated (collector bag, weighing dressings, etc.).

[6]Technical procedures: bladder voiding, manual exploration of the uterus, careful visual assessment of the lower genital tract. The audit defined lack of correspondence by the absence of any of these items from the protocol.

[7]Pharmacological procedures: plasma expansion by crystalloids, antibiotic prophylaxis after manual exploration of the uterus, uterotonic agents. The audit defined lack of correspondence by the absence of any of these items from the protocol.

[8]Immediate resuscitation of women: noninvasive monitoring (heart rate, blood pressure, pulse, and oximetry), establishment or securing of venous access, oxygen therapy, protection against hypothermia, and coagulation screens). The audit found no correspondence if any of these items was missing from the protocol.

[9]Relevant information about management and monitoring must be recorded on a specific monitoring sheet in the chart (paper or electronic).

[10]The audit defined concordance as the description of medical management of persistent PPH according to the French 2004 guidelines, as follows: if oxytocin fails to control the bleeding, sulprostone is to be administered by intravenous infusion (syringe).

[11]The audit defined concordance as the description of surgical management of persistent PPH according to the French 2004 guidelines as follows: conservative surgery techniques (arterial ligation and/or uterine compression suture) and, in case of failure, hysterectomy without salpingectomy.

Table S2. Incidence of PPH (> 500 mL), transfusion of packed red blood cells, surgical procedures, and artery embolization according to each audit criterion for PPH prevention.

Criteria that should be included in protocols	N=177[1]	Global PPH incidence[2] % (95% CI) n=101,339	*P*	Surgical procedures % (95% CI) n=3442	*P*	Blood transfusion % (95% CI) n=3442	*P*	Artery embolization % (95% CI) n=3442	*P*
**PPH definition[3]**									
Yes	51	3.6 (3.4, 3.8)	0.05	35.0 (32.1, 37.9)	<0.0001	12.3 (10.4, 14.4)	0.69	2.9 (2.0, 4.1)	-
No	126	3.3 (3.2, 3.5)		45.9 (43.8, 47.9)		12.8 (11.4, 14.2)		2.9 (2.2, 3.6)	
**Useful phone numbers[4]**									
Yes	56	3.6 (3.4, 3.8)	0.008	39.2 (36.6, 41.8)	0.001	14.1 (12.4, 16.1)	0.02	3.8 (2.9, 4.9)	0.007
No	121	3.3 (3.1, 3.4)		44.8 (42.6, 47.0)		11.5 (10.2, 13.0)		2.2 (1.6, 3.0)	
**Routine active management of the third stage of labor[5]**									
Yes	110	3.6 (3.5, 3.9)	<0.0001	42.2 (40.2, 44.2)	0.60	12.2 (10.9, 13.6)	0.25	3.1 (2.5, 3.9)	0.18
No	67	3.0 (2.8, 3.9)		43.1 (40.2, 46.1)		13.6 (11.6, 15.8)		2.3 (1.5, 3.4)	
**Time of PPH diagnosis recorded**									
Yes	78	3.5 (3.4, 3.7)	0.07	41.0 (39.6, 43.6)	0.06	13.3 (11.8, 14.9)	0.16	3.8 (3.0, 4.8)	0.004
No	99	3.3 (3.1, 3.5)		44.3 (41.8, 46.8)		11.7 (10.2, 13.4)		1.8 (1.2, 2.5)	
**Manual removal of the placenta indicated after 30 min[6]**									
Yes	84	3.3 (3.2, 3.5)	0.25	44.0 (41.5, 46.5)	0.10	13.3 (11.6, 15.0)	0.29	3.0 (2.2, 3.9)	0.82
No	93	3.5 (3.3, 3.6)		41.2 (39.0, 43.5)		12.1 (10.6, 13.6)		2.8 (2.1, 3.7)	

**Quantitative assessment of blood loss**[7]									
Yes	147	3.5 (3.3, 3.6)	0.04	41.6 (39.8, 43.4)	0.008	12.5 (11.3, 13.7)	0.46	3.1 (2.5, 3.8)	0.03
No	30	3.1 (2.9, 3.4)		48.2 (43.5, 52.8)		13.7 (10.7, 17.1)		1.3 (0.5, 2.8)	
**Volume of total blood loss recorded in a medical file**									
Yes	83	3.4 (3.2, 3.5)	0.46	39.9 (37.7, 42.2)	0.0007	13.6 (12.1, 15.3)	0.03	3.3 (2.5, 4.2)	0.11
No	94	3.5 (3.3, 3.6)		45.6 (43.1, 48.1)		11.4 (9.8, 13.1)		2.4 (1.7, 3.3)	

[1]Number of maternity units with the corresponding criterion among the 177 maternity unit protocols (6 without a local or regional protocol, for which each criterion was coded "no").

[2]PPH defined as > 500 mL.

[3]The audit defined agreement for the postpartum hemorrhage definition by two criteria: the presence of a specific definition, and its adherence to the definition in the French 2004 guidelines.

[4]The audit defined correspondence for "useful telephone numbers" by the presence of the numbers for the French Blood Agency and/or the hospital blood bank. If embolization was available onsite, the telephone number of the interventional radiology department also had to be listed. [5]The audit defined correspondence by the specification of the first-line pharmacologic product to be used, its dose, and the timing of its use.

[6]The audit considered that concordance was absent when the protocol did not specify manual removal of the placenta after 30 minutes in the absence of its spontaneous delivery.

[7]Regardless of how blood loss was estimated (collector bag, weighing dressings, etc.).

Table S3 Incidence of PPH (> 500 mL), transfusion of packed red blood cells, surgical procedures, and artery embolization, according to audit criteria for PPH management.

Criteria that should be included in the protocols	N= 177[1]	Global PPH incidence[2] % (95% CI) n=101,339	*P*	Surgical procedures % (95% CI) n=3442	*P*	Blood transfusion % (95% CI) n=3442	*P*	Radiologic artery embolization % (95% CI) n=3442	*P*
**Initial management**									
**Technical procedures**[3]									
Yes	136	3.4 (3.2, 3.5)	0.08	41.7 (39.9, 43.6)	0.07	13.2 (12.0, 14.5)	0.03	3.1 (2.5, 3.8)	0.08

No	41	3.6 (3.3, 3.9)		45.6 (41.8, 49.3)		10.2 (8.0, 12.7)		1.9 (1.0, 3.2)	
**Pharmacological procedures[4]**									
Yes	131	3.4 (3.3, 3.6)	0.15	42.2 (40.3, 44.0)	0.43	13.0 (11.8, 14.3)	0.19	3.1 (2.5, 3.9)	0.07
No	46	3.2 (3.0, 3.6)		43.8 (40.5, 47.6)		11.1 (8.9, 13.7)		1.9 (1.0, 3.2]	
**Immediate resuscitation[5]**									
Yes	82	3.6 (3.5, 3.8)	<0.0001	39.5 (37.3, 41.7)	<0.0001	12.7 (11.2, 14.2)	0.93	3.1 (2.4, 4.0)	0.40
No	95	3.1 (3.0, 3.3)		46.3 (43.8, 48.9)		12.6 (10.9, 14.3)		2.6 (1.9, 3.5)	
**PPH care included in a specific medical file[6]**									
Yes	71	3.6 (3.4, 3.8)	0.008	40.9 (38.5, 43.2)	0.05	14.0 (12.3, 15.7)	0.02	3.8 (2.9, 4.8)	0.002
No	106	3.3 (3.1, 3.4)		44.2 (41.8, 46.6)		11.3 (9.8, 12.8)		2.0 (1.4, 2.8)	
**Management for persistent PPH**									
**Medical management process described[7]**									
Yes	162	3.5 (3.4, 3.6)	<0.0001	42.3 (40.6, 44.0)	0.38	12.7 (11.6, 13.9)	0.38	2.9 (2.4, 3.6)	0.5
No	15	2.6 (2.2, 2.9)		45.5 (38.4, 52.7)		10.6 (6.7, 15.8)		2.0 (0.6, 5.1)	
**Surgical management process described[8]**									
Yes	155	3.5 (3.4, 3.6)	<0.0001	42.5 (40.8, 44.2)	0.97	12.6 (11.5, 13.8)	0.84	2.9 (2.4, 3.6)	0.5
No	22	2.3 (2.1, 2.7)		42.6 (36.1, 49.3)		12.2 (8.2, 17.1)		2.2 (0.1, 5.0)	

[1]Number of maternity units with the concordant criterion among the 177 maternity unit protocols (6 without a local or regional protocol, for which each criterion was coded 'no').
[2]PPH defined as > 500 ml.
[3]Technical procedures: bladder voiding, manual exploration of the uterus, careful visual assessment of the lower genital tract. The audit defined lack of correspondence by the absence of any of these items from the protocol.
[4]Pharmacological procedures: plasma expansion by crystalloids, antibiotic prophylaxis after manual exploration of the uterus, uterotonic agents. The audit defined lack of correspondence by the absence of any of these items from the protocol.
[5]Immediate resuscitation of the woman: noninvasive monitoring (heart rate, blood pressure, pulse, and oximetry), establishment or securing of venous access, oxygen therapy, protection against hypothermia, and coagulation screens). The audit found no correspondence if any of these items was missing from the protocol.
[6]Relevant information on management and monitoring must be recorded on a specific monitoring sheet in the chart (paper or electronic).

[7]The audit defined concordance as the description of medical management of persistent PPH according to the French 2004 guidelines, as follows: if oxytocin fails to control the bleeding, sulprostone is to be administered by intravenous infusion (syringe).
[8]The audit defined concordance as the description of surgical management of persistent PPH according to the French 2004 guidelines as follows: conservative surgery techniques (arterial ligation and/or uterine compression suture) and, in case of failure, hysterectomy without salpingectomy).
